# Supplementary material for: A UMLS-based spell checker for natural language processing in vaccine safety
Source: BMC Med Inform Decis Mak. 2007 Feb 12;7:3. doi: 10.1186/1472-6947-7-3 (PMC1805499; doi:10.1186/1472-6947-7-3)
Supplement: Additional file 13 — RAP application directory. Sets up directory for RAP (RDF application for PHP) source code [file 1472-6947-7-3-S13.gz › rap/doc/phpdoc/__filesource/fsource_default__vocabularyRDF.php.html]

xml version="1.0" encoding="iso-8859-1"?


File Source for RDF.php


# Source for file RDF.php

Documentation is available at RDF.php

```
1. <?php
2. // ----------------------------------------------------------------------------------
3. // Resource Description Framework (RDF) Vocabulary
4. // ----------------------------------------------------------------------------------
5. // Version                   : 0.9
6. // Authors                   : Daniel Westphal (dawe@gmx.de)
7. //
8. // Description               : Wrapper, defining resources for all terms of the
9. //                               Resource Description Framework (RDF).
10. //                               For details about RDF see: http://www.w3.org/RDF/.
11. //                                Using the wrapper allows you to define all aspects of
12. //                             the vocabulary in one spot, simplifing implementation and
13. //                             maintainence. Working with the vocabulary, you should use
14. //                             these resources as shortcuts in your code.
15. //
16. // ----------------------------------------------------------------------------------
17. // History:
18. // 02-21-2003                 : Initial version
19. // ----------------------------------------------------------------------------------


22. // RDF concepts (constants are defined in constants.php)

24. $RDF_Alt = new Resource(RDF_NAMESPACE_URI . RDF_ALT);
25. $RDF_Bag = new Resource(RDF_NAMESPACE_URI . RDF_BAG);
26. $RDF_Property = new Resource(RDF_NAMESPACE_URI . RDF_PROPERTY);
27. $RDF_Seq = new Resource(RDF_NAMESPACE_URI . RDF_SEQ);
28. $RDF_Statement = new Resource(RDF_NAMESPACE_URI . RDF_STATEMENT);
29. $RDF_List = new Resource(RDF_NAMESPACE_URI . RDF_LIST);
30. $RDF_nil = new Resource(RDF_NAMESPACE_URI . RDF_NIL);
31. $RDF_type = new Resource(RDF_NAMESPACE_URI . RDF_TYPE);
32. $RDF_rest = new Resource(RDF_NAMESPACE_URI . RDF_REST);
33. $RDF_first = new Resource(RDF_NAMESPACE_URI . RDF_FIRST);
34. $RDF_subject = new Resource(RDF_NAMESPACE_URI . RDF_SUBJECT);
35. $RDF_predicate = new Resource(RDF_NAMESPACE_URI . RDF_PREDICATE);
36. $RDF_object = new Resource(RDF_NAMESPACE_URI . RDF_OBJECT);
37. $RDF_Description = new Resource(RDF_NAMESPACE_URI . RDF_DESCRIPTION);
38. $RDF_ID = new Resource(RDF_NAMESPACE_URI . RDF_ID);
39. $RDF_about = new Resource(RDF_NAMESPACE_URI . RDF_ABOUT);
40. $RDF_aboutEach = new Resource(RDF_NAMESPACE_URI . RDF_ABOUT_EACH);
41. $RDF_aboutEachPrefix = new Resource(RDF_NAMESPACE_URI . RDF_ABOUT_EACH_PREFIX);
42. $RDF_bagID = new Resource(RDF_NAMESPACE_URI . RDF_BAG_ID);
43. $RDF_resource = new Resource(RDF_NAMESPACE_URI . RDF_RESOURCE);
44. $RDF_parseType = new Resource(RDF_NAMESPACE_URI . RDF_PARSE_TYPE);
45. $RDF_Literal = new Resource(RDF_NAMESPACE_URI . RDF_PARSE_TYPE_LITERAL);
46. $RDF_Resource = new Resource(RDF_NAMESPACE_URI . RDF_PARSE_TYPE_RESOURCE);
47. $RDF_li = new Resource(RDF_NAMESPACE_URI . RDF_LI);
48. $RDF_nodeID = new Resource(RDF_NAMESPACE_URI . RDF_NODEID);
49. $RDF_datatype = new Resource(RDF_NAMESPACE_URI . RDF_DATATYPE);
50. $RDF_seeAlso = new Resource(RDF_NAMESPACE_URI . RDF_SEEALSO);


54. ?>
```

Documentation generated on Fri, 17 Dec 2004 16:16:51 +0100 by phpDocumentor 1.3.0RC3
